# Comparative evaluation of mouthwashes containing propolis and chlorhexidine for controlling inflammation resulting from plaque accumulation in patients with chronic gingivitis

**DOI:** 10.34172/japid.2023.014

**Published:** 2023-08-29

**Authors:** Ashkan Salari, Masoumeh Nikkhah, Azita Alamzadeh

**Affiliations:** ^1^Dental Sciences Research Center, Department of Periodontics, School of Dentistry, Guilan University of Medical Sciences, Rasht, Iran; ^2^Periodontist, Rasht, Iran; ^3^Dentist, Rasht, Iran

**Keywords:** Dental plaque, Gingival index, Gingivitis, Inflammation, Propolis

## Abstract

**Background.:**

Limited data are available on the effect of mouthwashes containing Iranian propolis on plaque index (PI) and gingival index (GI) in patients with chronic gingivitis. The present study compared the effects of propolis and chlorhexidine (CHX) mouthwashes in patients with chronic gingivitis due to plaque accumulation.

**Methods.:**

In the present interventional study, 28 patients 18‒50 years of age with generalized chronic gingivitis were assigned to two groups (n=14). Periodontal parameters, including PI and GI, were determined in all the subjects at baseline. Groups A and B received CHX and propolis mouthwashes, respectively. All the subjects used the mouthwashes for two weeks. Then all the parameters were evaluated gain. Independent t-test was used to compare the periodontal parameters between the two groups. Paired t-test was used for intra-group comparisons. Statistical significance was defined at *P*<0.05

**Results.:**

Two weeks after using the mouthwashes, the mean PI in the CHX group (21.71±1.63) was significantly lower than that in the propolis group (33.91±5.96). However, the mean PI and GI in the propolis group decreased significantly compared to the baseline (*P*=0.00).

**Conclusion.:**

Propolis significantly decreased the mean plaque and gingival inflammation in patients with chronic gingivitis. Although the reduction in PI in the propolis group was a little less than in the CHX group, the efficacy of propolis in reducing GI was comparable to CHX.

## Introduction

 Dental plaque-associated gingivitis is the most prevalent gingival disease. Dental plaque is the primary etiologic factor for gingivitis.^1^ Plaque formation on the tooth surface is a dynamic and regular process that begins with the adhesion of plaque-forming bacteria to tooth surfaces. Microbial biofilm aggregation on tooth surfaces results in an inflammatory process in the surrounding gingival tissues. As long as the microbial biofilm is present in the vicinity of gingival tissues, local inflammation will persist and will only resolve after completely removing the biofilm.^2,3^

 Currently, the primary preventive measure for gingivitis and periodontitis relies on the adequate elimination of plaque. The use of toothpaste and toothbrush is possibly the most common and potentially the most effective method to remove dental plaque in developed counties.^4,5^ However, despite the emphasis on oral hygiene measures, gingivitis is highly prevalent, and it is necessary to use tools and factors that do not rely much on the patients’ skills and compliance. Therefore, chemical agents have been introduced to control plaque.^6^

 Chlorhexidine (CHX) digluconate is a bisguanidine antiseptic recognized as the most effective antiseptic to inhibit plaque and prevent gingivitis. This chemical agent has been successful in combating plaque and gingivitis to some extent. It is a substitute and not just a supplementary oral hygiene method. Despite the high anti-plaque and anti-gingivitis effects of CHX, it has some side effects, including brown discoloration of teeth, some restorations, and the dorsum of the tongue, a change in taste perception, and a bitter taste in the oral cavity, oral mucosal erosion, an increase in the formation of supragingival calculus, and in some cases, unilateral or bilateral swelling of the parotid gland. The side effects of using this product have resulted in extensive research to introduce new products with the same quality and power, with minimum side effects.^7,8^

 Propolis is a natural resin product with a herbal origin, which honeybees use as a sealant for the spaces and cracks of beehives. The color and chemical structure of propolis are different depending on the resin sources found around the beehive.^9^ The chemical structure of propolis consists of resin ingredients, honeybee wax, ether, aromatic oils, and 5% honeybee pollen. Propolis is rich in vitamins A, B_1_, B_2_, biotin, and bioflavonoids. Antimicrobial activity is one of the most important properties of propolis. To date, the strong inhibitory effect of propolis has been reported on 21 bacterial species, 9 fungal specie, 3 protozoon species, and a wide array of viruses. The antibacterial activity of propolis is attributed to flavonoids, aromatic acids, and esters in the structure of the resin.^10,11^

 The antibacterial properties of propolis extract have been shown against *Streptococcus mutans*, mainly in the oral cavity. It has a role in the formation of dental plaque. Several studies have shown the therapeutic effects of propolis on resolving gingivitis and oral cavity lesions. One of its main applications might be improving oral hygiene and decreasing dental plaque and gingival inflammation with minimal side effects.^12-15^

 Considering differences in the chemical compositions of propolis in different geographic locations and a lack of information on the oral rinse form of propolis, the present study compared the anti-plaque and anti-inflammatory properties of propolis mouthwash in patients with chronic gingivitis with those of CHX gluconate in an Iranian population.

## Methods

 The present interventional study was conducted in the Department of Periodontics, Faculty of Dentistry, Guilan University of Medical Sciences in 2019‒2020. The participants were randomly assigned to the study groups (group A: CHX mouthwash and group B: propolis mouthwash) based on the inclusion criteria: patients 18‒50 years of age with chronic generalized gingivitis, the presence of at least 20 teeth in the oral cavity, no use of any mouthwashes in the past two weeks, no use of antibiotics in the past two weeks, absence of periodontitis, dental caries, and unrated dental caries, absence of soft and hard tissue lesions, no use of any orthodontic appliances and fixed prostheses, no systemic diseases compromising the immune system, including diabetes and AIDS.^16^

 The following patients were excluded from the study: patients not attending the follow-up sessions during the study, those taking antibiotics during the study, and those not able to follow the recommended oral hygiene measures.^16^

 Twenty-eight patients with generalized chronic gingivitis were enrolled using a simple sampling method by gradual referrals after signing informed consent forms following proper explanations about the study procedures and randomly assigned to groups A, CHX 0.2% (Irsha Co, Iran) and B, propolis 30% extracts (Mashhad Tak Toos Soren Co, Iran). The patients underwent an initial clinical examination with a dental minor and a Williams periodontal probe (JUYA, Pakistan) by a periodontist. The O’Leary plaque index (PI) and Löe and Silness gingival index (GI) parameters were determined and recorded. The patients were provided with the necessary recommendations concerning diet (regarding the formation and adhesion of plaque) and oral hygiene measures, including flossing their teeth once a day and brushing them with the Bass technique for 5 minutes twice daily.

 Each patient was given a similar bottle of mouthwash without a label and asked to gargle 10 mL of the mouthwash for 60 seconds twice daily for two weeks. The plaque and gingival indices were determined and recorded again to compare and identify changes.

 The data were analyzed with SPSS 22, using paired t-test and independent t-test for intra-group and inter-group comparisons, respectively, at a significance level of *P*< 0.05.

## Results

 Twenty-eight patients participated in the present study in two groups: A (n = 14) and B (n = 14). The mean PI in groups A and B were 90.64 ± 8.65 and 92.5 ± 6.85, respectively, at baseline. The mean GI in groups A and B were 1.42 ± 0.85 and 1.57 ± 0.75, respectively, at baseline, with no significant difference between the two groups (Table 1).

**Table 1:** Comparison of periodontal clinical parameters (PI and GI) in groups A and B before and two weeks after using the mouthwashes

		**Group A** **(mean±SD)**	**Group B** **(mean±SD)**	***P *** **value**
Before mouthwashes	PI	90.64 ± 8.65	92.50 ± 6.85	0.41
	GI	1.42 ± 0.85	1.57 ± 0.75	0.41
After mouthwashes	PI	21.71 ± 1.63	33.21 ± 5.96	0.00*
	GI	0.21 ± 0.42	0.35 ± 0.49	0.1

*Significant.

 The PI decreased in both groups after using the mouthwashes; however, its mean in group A (21.71 ± 1.63) was significantly less than that in group B (33.21 ± 5.96) (*P* = 0.00). Therefore, CHX mouthwash decreased the PI significantly compared to propolis mouthwash.

 The GI, too, decreased in both groups after the intervention; however, there was no significant difference between groups A (0.42 ± 0.21) and B (0.49 ± 0.35) (*P* = 0.1). Therefore, despite the decrease in the GI in both groups after using the mouthwashes, there were no significant differences between CHX and propolis in this respect (Table 1).

**Figure 1:**
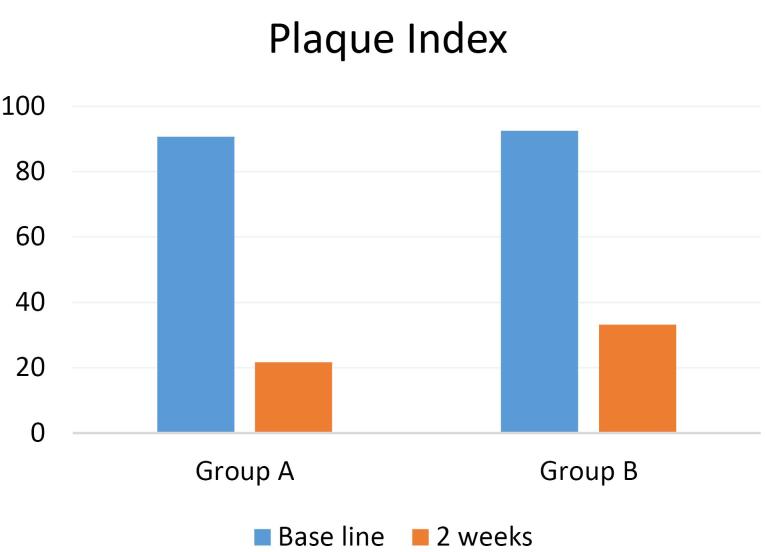
Comparison of the means of plaque index between the study groups before and two weeks after using the mouthwashes.

**Figure 2:**
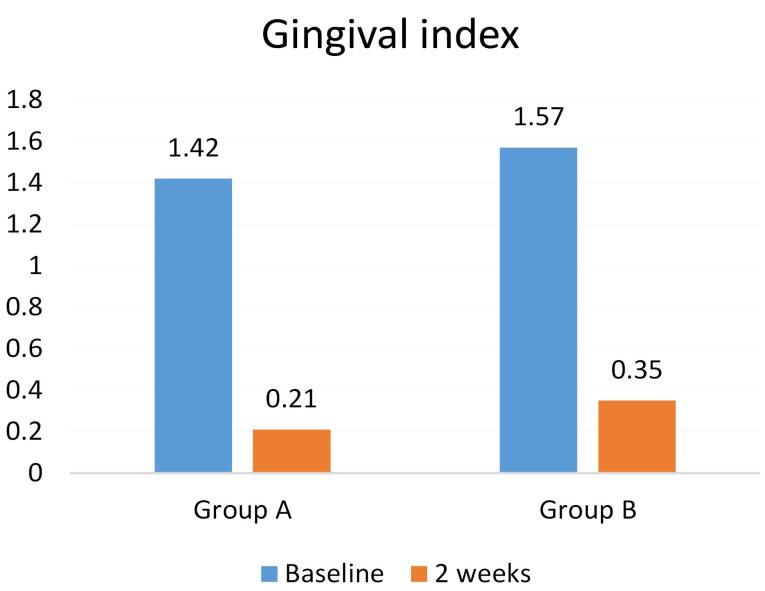
Comparison of the means of gingival index between the study groups before and two weeks after using the mouthwashes.

 In both groups, using CHX and propolis mouthwashes decreased Pi and GI. In addition, both mouthwashes significantly decreased these two indexes compared to baseline (*P* = 0.00) (Table 2; Figures 1 and 2).

**Table 2:** Comparison of periodontal clinical parameters in groups A and B before and two weeks after using mouthwashes

		**Before mouthwash** **(mean±SD)**	**After mouthwash** **(mean±SD)**	**Mean difference**	***P*** ** value**
Group A	PI	90.64 ± 8.65	21.71 ± 1.63	68.92	0.00*
	GI	1.42 ± 0.85	0.21 ± 0.42	1.21	0.00*
Group B	PI	85.6 ± 8.92	33.21 ± 5.96	59.28	0.00*
	GI	1.57 ± 0.75	0.35 ± 0.49	1.21	0.00*

*Significant.

## Discussion

 The present interventional study compared propolis and CHX digluconate mouthwashes to control plaque-induced gingivitis.

 Dental plaque is the etiologic agent of gingivitis. Supragingival plaque control relies on the regular removal of plaque with routine mechanical methods such as toothbrushing and the use of dental floss. Chemical factors to control plaque, including mouthwashes, have attracted attention due to differences in individuals’ ability to control plaque and apply oral hygiene instructions. CHX has been reported to be the most effective antiseptic to inhibit plaque and prevent gingivitis; however, it can only be considered an adjunctive treatment to replace other plaque control methods. CHX is a cationic bisguanidine with strong antibacterial properties, which increases bacterial cell membrane permeability, resulting in the leakage of intracellular components and microorganism death.^3-5^

 CHX is easily attached to different surfaces, including the pellicle, due to its cationic properties and exhibits bacteriostatic activity for over 12 hours, contrary to other antiseptic agents. However, despite these unique properties of CHX, its long-term use is limited due to its initial bitter taste and side effects, such as tooth staining and a change in the gustatory sense. In this context, attention has shifted toward producing new products, especially natural and plant-derived ones.^7^

 Therefore, new products, especially natural and plant-derived products, which have the properties of CHX without its complications, are advocated. In the present study, the clinical effects of propolis on controlling gingivitis were compared with those of 0.2% CHX. Propolis is a resin material collected from tree trunks, flower nectar, or other plant sources by honeybees.

 In the present study, in comparison of PI and GI of the whole oral cavity before and after using 0.2% CHX mouthwash and propolis mouthwash, both mouthwashes significantly reduced the mean PI and G, consistent with studies by Savita et al,^17^ Arjun et al,^18^ Dodwad and Kukreja,^19^ and Krishna et al.^20^

 According to the results of our study, in comparison of 0.2% CHX mouthwash with propolis mouthwash in terms of the effect on PI, both mouthwashes reduced the PI, but CHX was better than propolis, similar to the results reported by Murray et al^21^ and Dodwad and Kukreja.^19^ However, Krishna et al^20^ and Arjun et al^18^ showed that propolis was significantly better than CHX in reducing plaque accumulation. Also, Savita et al,^17^ Porwal et al,^22^ and Santiago et al^23^ concluded that propolis reduced the PI similar to CHX. The reasons for this difference may be the type of propolis mouthwash (different extracts) and the study method (number of times the mouthwash is used and the study duration).

 Another result of our study, in comparing the mean changes of the GI after using mouthwashes, propolis reduced the GI similar to CHX, and no significant difference was found, consistent with the results of the study by Savita et al.^17^ Contrary to our results, studies by Dodwad and Kukreja,^19^ Arjun et al,^18^ Krishna et al,^20^ and Porwal et al^22^ showed that propolis reduced GI significantly better than CHX, which might be explained by the effect of regional flora on honeybee nutrition and the production of propolis with different compositions and effects.

 Anauate-Netto et al^16^ compared the effects of propolis and 0.12% CHX mouthwashes on gingivitis and reported that propolis was superior to CHX in reducing mean papillary bleeding index. Nevertheless, this index was not evaluated in the present study.

 In the present study, although the anti-plaque effect of propolis was slightly less than CHX, propolis was comparable to CHX in reducing gingival inflammation. Propolis has anti-inflammatory properties, inhibits the production of prostaglandins, and results in a rapid reduction in tissue inflammation.^24,25^

 In previous studies, no side effects were observed in patients following the use of propolis mouthwash. One of the most common side effects of the clinical use of CHX is the discoloration of the teeth, which causes dissatisfaction among patients. Due to the lack of tooth discoloration, propolis can be considered a safe alternative to CHX for reducing plaque and gingival inflammation in patients for daily use.^17,19-23^ Further studies are necessary considering the limitations of the study population, the duration of intervention, and a paucity of studies on the effect of propolis on the periodontium.

## Conclusion

 According to the present study, CHX and propolis mouthwashes significantly decreased the PI and GI in patients with chronic gingivitis. CHX was significantly more effective than propolis in reducing the PI. However, there were no significant differences between the two mouthwashes concerning the GI. Therefore, considering the side effects of the long-term use of CHX, propolis might be regarded as a natural and safe alternative for CHX to control plaque and gingivitis with no side effects for daily use.

## Competing Interests

 The authors declare no competing interests concerning the authorship and/or publication of this paper.

## Data Availability Statement

 The raw data from the reported study are available upon request from the corresponding author.

## Ethical Approval

 The study protocol was approved by the Ethics Committee of Guilan University of Medical Sciences under the code IR.GUMS.REC.1398.454.

## Funding

 None.
